# Reduced Quadriceps Motor-Evoked Potentials in an Individual with Unilateral Knee Osteoarthritis: A Case Report

**DOI:** 10.1155/2011/537420

**Published:** 2011-09-29

**Authors:** Michael A. Hunt, Jeanie R. Zabukovec, Sue Peters, Courtney L. Pollock, Meghan A. Linsdell, Lara A. Boyd

**Affiliations:** Department of Physical Therapy, University of British Columbia, Vancouver, BC, Canada V6T 1Z3

## Abstract

One male with unilateral osteoarthritis (OA) of the knee underwent testing of corticospinal (CS) excitability (as quantified from motor-evoked potentials (MEPs) in the rectus femoris (RF) using transcranial magnetic stimulation) and quadriceps muscle strength. Baseline data indicated reduced MEP amplitudes in the RF of the affected limb compared to the unaffected limb. Increases in RF MEP amplitudes from both limbs were observed immediately following a 30-minute exercise session focusing on muscle strengthening. Following an 8-week muscle strengthening intervention, the participant exhibited increased MEP amplitudes and muscle strength in the affected limb. These findings suggest that alterations in peripheral muscle function found in patients with knee OA may have an origin centrally within the motor cortex and that interlimb differences may be evident in those with unilateral disease. These findings also suggest that CS excitability may be improved following a muscle strengthening intervention.

## 1. Introduction

Osteoarthritis (OA) is a prevalent chronic condition often associated with symptoms of pain and physical dysfunction. Biomechanical studies in this patient population have typically focused on the measurement of knee joint load obtained from three-dimensional gait analysis and/or methods of assessing neuromuscular function. Altered or excessive joint loading is a recognized risk factor for the degradation of articular cartilage [1, 2], and biomechanical studies have identified joint-specific measures of load during movement, that are predictive of disease progression. For example, the magnitude of the external knee adduction moment (KAM) measured during walking—an indirect measure of dynamic load in the medial compartment of the knee joint [3]—has been shown to be a strong predictor of medial compartment tibiofemoral knee OA progression [4]. 

In addition to producing movement, the muscles of the lower limb are believed to play a role in the dissipation of load away from articular cartilage in the knee joint [5]. However, patients with knee OA often exhibit strength deficiencies [6–8] and altered neuromuscular control [9] compared to those without OA, which may result in suboptimal load dissipation within the knee. Electromyographical studies during walking have also shown increased muscle cocontraction in those with knee OA [10, 11], indicative of altered motor output. Due to these differences in neuromuscular outcomes, many researchers and clinicians have attempted to restore muscle strength and function in those with knee OA using a variety of treatment approaches ranging from simple muscle strengthening programs to complex neuromuscular retraining interventions, all with varying effects on muscle and joint function.

Though the motor output changes resulting from knee OA have been well-studied, the central contribution to muscle activity is less well known. Noninvasive activation of the motor cortex using transcranial magnetic stimulation (TMS) to evoke responses in peripheral muscles (termed motor evoked potentials (MEPs)) provides a reliable and accurate method of assessing corticospinal (CS) excitability and the contribution of central structures to muscle activity [12]. This method has been used for a number of patient populations ranging from those with neurological impairments including stroke [12] to orthopaedic involvement such as those with muscle weakness [13], knee pain [14], or anterior cruciate ligament injury [15]. We are unaware of any studies that have used TMS to study CS excitability and central contributions to muscle activity in patients with knee OA. As a result, the purpose of the present study was to assess CS excitability and quadriceps muscle strength in an individual with unilateral knee OA before and after a muscle strengthening program.

## 2. Case Presentation

A 67-year-old, right leg dominant male (1.63 m; 61.4 kg) underwent physical assessment pertaining to reports of unilateral left knee pain and muscle weakness. He first experienced moderate left knee pain five months previously when attempting to carry a heavy object up a flight of stairs. There were no reports of previous injuries to the feet, knees, hips, or back that required medical attention. The pain was self-managed using anti-inflammatory medication and glucosamine with chondroitin, however he still reported a moderate (4/10) amount of knee pain on most days. He was able to ambulate unaided, but occasionally used a cane when walking longer distances. Radiographs confirmed the presence of mild OA in the left knee with definite medial joint space narrowing and one small medial tibial osteophyte (Kellgren and Lawrence grade 2 [16]). Radiographs of the right knee were unremarkable. Clinical examination did not reveal any ligamentous laxity or patellofemoral involvement, or any observable difference in knee circumference or thigh muscle girth. In accordance with established and institutional safety guidelines for the use of TMS, the participant was screened for history of seizure, medication use, metal implants in the brain or head, and history of neurological diagnosis prior to inclusion in this study [17, 18].

**Figure 1:**
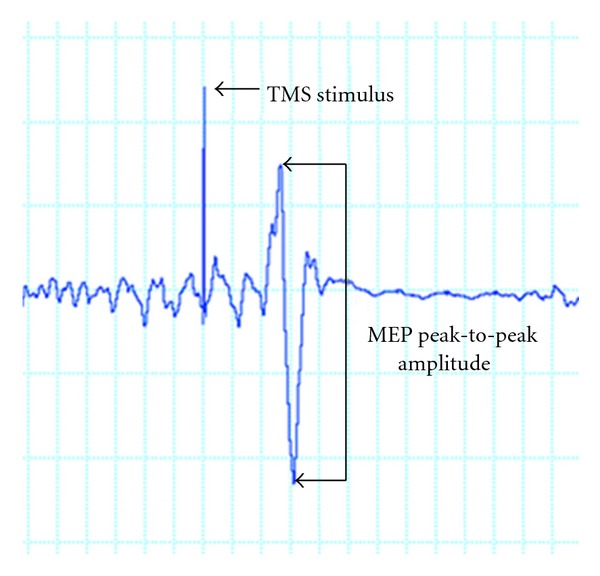
MEP magnitude was determined by identifying the peak-to-peak amplitude of the quadriceps EMG trace following each TMS-evoked stimulus.

Assessment of neuromuscular function included measurement of CS excitability (as quantified using MEPs) and isometric muscle strength. CS excitability was assessed using TMS with a 70 mm double cone coil (Magstim Super Rapid, Magstim Company, Ltd.). Surface bipolar Ag-AgCl electrodes (10 mm diameter) were placed 25 mm apart on the belly of the left and right rectus femoris (RF), and a ground electrode was placed on the patella. The skin was shaved, cleaned, and abraded prior to application of the electrodes to reduce electrical impedance. During stimulation, the coil trajectory that elicited the best MEP for the RF for each leg was marked using BrainSight software (Rogue, Montreal) and stored for future reference, and the marker for the RF trajectory was placed on a template MRI brain image. To reduce both intra- and intersession variability in the application of TMS, the same reference brain and RF trajectory marker were used in each TMS mapping time point. Active motor threshold (AMT) was determined while the participant maintained a low level contraction and defined as the lowest stimulator intensity that generated 5 MEPs across 10 trials, each with a peak-to-peak amplitude (Figure 1) of at least 200 *μ*V. MEPs were then elicited from the motor cortex at stimulus intensities of 105%, 115%, 120%, 125%, 130%, 135%, and 145% AMT. A stimulus response (motor recruitment) curve [19] was generated for each limb by calculating the average peak MEP magnitude from ten consecutive stimuli at each intensity, with approximately 1 sec to 4 sec between each stimulus. To account for the possibility of baseline variability, data from each stimulus intensity were normalized to 105% AMT for each stimulation session. Linear regression was then used to compute the slope parameter for the curve, as suggested previously [15, 20]. This procedure was repeated immediately following a 30-minute exercise session, using the same electrode placement and coil trajectory. This exercise session was intended to familiarize the participant with a home-based exercise program and included unilateral (affected limb only) open-kinetic chain knee extension in sitting, knee flexion in standing, and hip abduction in standing and side lying. Closed kinetic chain exercises include half-squats against a wall, forward lunges, and seated leg presses (not part of the home program due to equipment requirements), all involving both limbs. Resistance, when applicable, was provided from cuff weights attached around the ankle and chosen based on the ability to perform 10 repetitions with moderate difficulty. 

On a separate visit to the laboratory within the same week as the assessment of CS excitability, maximal isometric muscle strength was measured while the participant was seated in an isokinetic dynamometer (Biodex System 4; Biodex Medical Systems Inc., Shirley, NY). Maximal isometric knee extension torque from each limb was measured with the hip in 90° of flexion and the knee in 30° of flexion. After a warm-up trial for the purposes of equipment familiarization, three isometric trials of five-second duration were completed for each limb. The maximum torque exerted onto the dynamometer from the three trials was identified and normalized to body mass (Nm/kg).

Following baseline muscle strength testing, the participant was prescribed the series of six muscle-strengthening exercises described above and instructed to complete each exercise for 3 sets of 10 repetitions at home on at least four days per week over 8 weeks. Ankle cuff weights were provided for resistance to the seated knee extension, side lying hip abduction, as well as the standing knee flexion, and hip abduction. The participant met with a physiotherapist four times over this period to ensure proper exercise performance and safe progression of resistance, and to perform additional exercises (e.g., seated leg press and standing hip adduction with cable resistance) not possible as part of the home program. CS excitability (using the same electrode placement and coil trajectory from baseline, AMT was determined again) and muscle strength were assessed again at the end of the 8-week muscle strengthening intervention, using the same techniques with the isokinetic dynamometer and outcomes previously described. Finally, overall average knee pain was assessed before and after the strengthening intervention, using an 11-point numerical rating scale with 0 representing “no pain” and 10 representing “worst pain imaginable”. 

**Figure 2:**
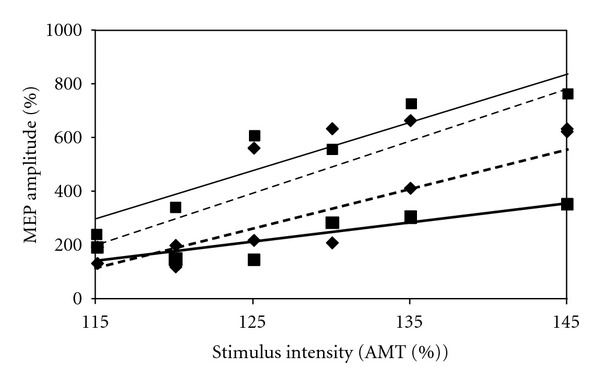
Stimulus response curves at the baseline testing session produced by calculating average MEP magnitudes at TMS stimulus intensities equal to 115%, 120%, 125%, 130%, 135%, and 145% of the active motor threshold (AMT) for each limb. Values are represented as a normalized percentage of the average MEP amplitude calculated at 105% of the AMT for the affected limb (squares and solid lines) and unaffected limb (diamonds and dashed lines). Thick lines correspond to the first baseline testing session (before the initial 30-minute exercise session), while thin lines correspond to the second baseline testing session (immediately following the initial 30-minute exercise session).

**Figure 3:**
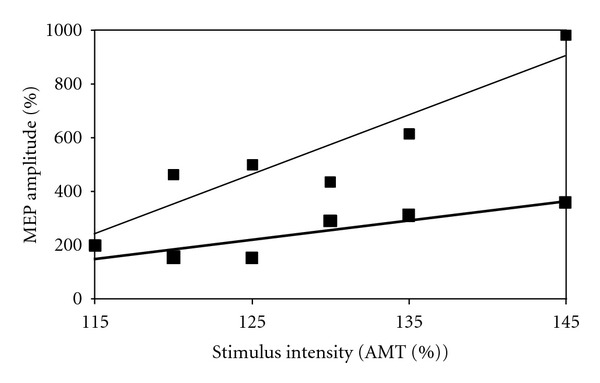
Stimulus response curves for the affected limb from the baseline, preexercise testing session (thick line) and 8-week follow-up testing session (thin line) produced by calculating average MEP amplitudes at TMS stimulus intensities equal to 115%, 120%, 125%, 130%, 135%, and 145% of the active motor threshold (AMT). Values are represented as a normalized percentage of the average MEP magnitude calculated at 105% of the AMT from each testing session.

Between-limb differences in MEP amplitude were evident across all stimulus intensities at baseline (Figure 2). In general, RF MEPs were higher in the unaffected limb compared to the affected limb across all stimulus intensities prior to 30 minutes of exercise. Following the 30-minute exercise familiarization session, MEP amplitudes in both limbs increased at all stimulus intensities, with the exception of 120% and 145% AMT in the unaffected limb. Differences between limbs in the postexercise session were small at all stimulus intensities. Following 8 weeks of lower limb muscle strengthening, MEP amplitudes in the affected limb increased, compared to baseline at all stimulus intensities except 115% of AMT (Figure 3). Finally, overall knee pain was reduced from 4/10 to 0/10, and maximal isometric quadriceps torque increased from 1.25 Nm/kg to 1.60 Nm/kg following the 8-week strengthening intervention.

## 3. Discussion

Results from this case study show that interlimb differences in CS excitability existed in an individual with unilateral OA of the knee. Specifically, CS excitability was reduced in the RF muscle crossing the knee affected with OA, compared to the contralateral, unaffected limb. Further, increases in CS excitability in the hemisphere controlling the affected limb were evident immediately following a single muscle strengthening exercise session as well as after completing an 8-week intervention focusing on improving muscle strength. Taken together, these findings indicate the likelihood of alterations along the CS tract in individuals with knee OA, as well as the possibility of improving motor function with exercise training.

Though there are no data in the literature reporting cortical excitability in individuals with knee OA, there are previous examinations of patients with other knee pathologies. On et al. [14] reported increases in MEP magnitudes in the vastus medialis obliques and vastus lateralis muscles in a cohort of younger (ages 23 to 37 years) individuals with patellofemoral pain syndrome compared to healthy controls. Héroux and Tremblay [15] identified three unique subgroups of patients with anterior cruciate ligament injures based on their interlimb differences in RF MEP amplitudes. Of the 10 participants, three exhibited steeper stimulus response curves (i.e., higher MEPs across all stimulus intensities) in their injured limb compared to the uninjured; four participants exhibited steeper stimulus response curves in the uninjured limb, while the remaining three participants exhibited symmetry between the two limbs. They postulated that these differences in MEP presentation were the result of impairments in quadriceps activation in some individuals and distinguished between “copers” and “noncopers” known to exist in this patient population that reflect either an ability to exhibit near normal biomechanics and joint function despite the injury, or an increasing risk of reinjury and functional decline [21]. Identification of similar subgroups in individuals with knee OA would have important implications for the management of the disease given that proper muscle function is believed to play an important role in cartilage offloading during movement. Thus, identification of those who exhibit impairments in cortical excitability and motor output would permit more focused rehabilitation and improvements in subsequent joint protection. In contrast, those without cortical and motor impairments may not necessarily require muscle reconditioning approaches and alternate treatment strategies could be provided.

The individual in the current case study exhibited increases in MEP amplitude following 8 weeks of muscle strengthening exercises. Results from previous studies examining the effect of muscle strengthening on MEP amplitudes point to differences in outcomes based on the muscle being examined. Strengthening exercises have not been shown to increase MEP amplitude from the flexor digitorum indices muscle [22, 23] nor the biceps brachii muscle [24], but have been shown to increase MEP amplitudes from the soleus muscle [25]. Modulation of MEPs is also dependent on the functional implication of muscles in the task. Schieppati et al. [26] demonstrated an increase in MEPs in prime movers in precision tasks requiring control compared to power tasks, despite comparable EMG levels. Jensen and colleagues [24] also demonstrated that several weeks of skill training induced CS excitability, whereas strength training induced a decrease. Thus, it may be that the MEP response to exercise is not only dependent on the muscle group, but the biomechanical requirements of the muscles for movement. 

The increases in MEP amplitudes from the RF of the affected limb in the present study suggest some form of plasticity in the motor system of the participant that may have occurred due to improved neuromuscular efficiency, cortical changes resulting from decreased pain responses, or both. Importantly, increases in MEP magnitude were evident following a single 30-minute exercise session. This may have been the result of upregulation of central nervous system, a general improvement in activation of the quadriceps with force production compared to the preexercise, sedentary situation, or a reduction in muscle inhibition after exercise due to decreased joint pain. Indeed, the term “arthrogenic muscle inhibition” has been used to describe a diminished ability to contract a muscle following an injury [27], which may reduce the excitability of motor neurons and subsequent motor output [28]. Many authors suggest that these changes are manifested at the cortical level to produce deficits in strength following orthopaedic injuries [29–32], while others suggest that these changes in cortical excitability result from prolonged muscle activation and movement impairments [33, 34]. Regardless, a viscous cycle of pain and movement restrictions—common in those with knee OA—may result in impairments in CS excitability and motor output, causing further movement restrictions and subsequent joint pain. Given the suggested role of muscle in OA pathogenesis [5], identification of methods to enhance neuromuscular efficiency while simultaneously reducing joint pain represents an important clinical and research objective for this patient population. That said, the limited ability to draw firm conclusions of the relationship between OA and cortical excitability and resultant MEPs from a single patient must be acknowledged. Further work in this area is warranted to better understand these relationships, with an aim to improve the clinical management of the disease.

## 4. Conclusions

These findings show that CS excitability and subsequent RF MEPs are different based on the presence or absence of knee OA within an individual. It was also shown that increases in MEP amplitudes can be achieved with short-term (30-minute) and longer-term (8-week) exercise. Given the continued use of strengthening exercises in clinical management of knee OA as well as the recent focus on the use of neuromuscular retraining programs for knee OA, continued research is needed to determine the optimal methods of restoring neuromuscular function in this patient population. Further, an understanding of the role of reduced CS excitability and MEPs on measures of joint loading during movement—a known risk factor for OA progression—is needed to better understand disease pathology and develop effective treatments focusing on improving muscle and joint function.

## References

[1] Radin EL, Paul IL (1971). Response of joints to impact loading. I. In vitro wear. *Arthritis and Rheumatism*.

[2] Radin EL, Parker HG, Pugh JW, Steinberg RS, Paul IL, Rose RM (1973). Response of joints to impact loading III. Relationship between trabecular microfractures and cartilage degeneration. *Journal of Biomechanics*.

[3] Schipplein OD, Andriacchi TP (1991). Interaction between active and passive knee stabilizers during level walking. *Journal of Orthopaedic Research*.

[4] Miyazaki T, Wada M, Kawahara H, Sato M, Baba H, Shimada S (2002). Dynamic load at baseline can predict radiographic disease progression in medial compartment knee osteoarthritis. *Annals of the Rheumatic Diseases*.

[5] Bennell KL, Hunt MA, Wrigley TV, Lim BW, Hinman RS (2008). Role of muscle in the genesis and management of knee osteoarthritis. *Rheumatic Disease Clinics of North America*.

[6] Hinman RS, Hunt MA, Creaby MW, Wrigley TV, McManus FJ, Bennell KL (2010). Hip muscle weakness in individuals with medial knee osteoarthritis. *Arthritis Care and Research*.

[7] Messier SP, Loeser RF, Hoover JL, Semble EL, Wise CM (1992). Osteoarthritis of the knee: effects on gait, strength, and flexibility. *Archives of Physical Medicine and Rehabilitation*.

[8] Slemenda C, Brandt KD, Heilman DK (1997). Quadriceps weakness and osteoarthritis of the knee. *Annals of Internal Medicine*.

[9] Hortobágyi T, Garry J, Holbert D, Devita P (2004). Aberrations in the control of quadriceps muscle force in patients with knee osteoarthritis. *Arthritis Care and Research*.

[10] Astephen JL, Deluzio KJ, Caldwell GE, Dunbar MJ, Hubley-Kozey CL (2008). Gait and neuromuscular pattern changes are associated with differences in knee osteoarthritis severity levels. *Journal of Biomechanics*.

[11] Hubley-Kozey CL, Deluzio KJ, Landry SC, McNutt JS, Stanish WD (2006). Neuromuscular alterations during walking in persons with moderate knee osteoarthritis. *Journal of Electromyography and Kinesiology*.

[12] Cacchio A, Paoloni M, Cimini N (2011). Reliability of TMS-related measures of tibialis anterior muscle in patients with chronic stroke and healthy subjects. *Journal of the Neurological Sciences*.

[13] Urbach D, Berth A, Awiszus F (2005). Effect of transcranial magnetic stimulation on voluntary activation in patients with quadriceps weakness. *Muscle and Nerve*.

[14] On AY, Uludağ B, Taşkiran E, Ertekin C (2004). Differential corticomotor control of a muscle adjacent to a painful joint. *Neurorehabilitation and Neural Repair*.

[15] Héroux ME, Tremblay F (2006). Corticomotor excitability associated with unilateral knee dysfunction secondary to anterior cruciate ligament injury. *Knee Surgery, Sports Traumatology, Arthroscopy*.

[16] Kellgren JH, Lawrence JS (1957). Radiological assessment of osteo-arthrosis. *Annals of the Rheumatic Diseases*.

[17] Wassermann EM (1998). Risk and safety of repetitive transcranial magnetic stimulation: report and suggested guidelines from the International Workshop on the Safety of Repetitive Transcranial Magnetic Stimulation, June 5-7, 1996. *Electroencephalography and Clinical Neurophysiology*.

[18] Rossi S, Hallett M, Rossini PM, Pascual-Leone A (2009). Safety, ethical considerations, and application guidelines for the use of transcranial magnetic stimulation in clinical practice and research. *Clinical Neurophysiology*.

[19] Ridding MC, Rothwell JC (1997). Stimulus/response curves as a method of measuring motor cortical excitability in man. *Electroencephalography and Clinical Neurophysiology*.

[20] Ray J, McNamara B, Boniface S (2002). Acquisition and expression of proximal and distal upper limb stimulus-response curves to transcranial magnetic stimulation. *Muscle and Nerve*.

[21] Chmielewski TL, Stackhouse S, Axe MJ, Snyder-Mackler L (2004). A prospective analysis of incidence and severity of quadriceps inhibition in a consecutive sample of 100 patients with complete acute anterior cruciate ligament rupture. *Journal of Orthopaedic Research*.

[22] Kidgell DJ, Pearce AJ (2010). Corticospinal properties following short-term strength training of an intrinsic hand muscle. *Human Movement Science*.

[23] Carroll TJ, Riek S, Carson RG (2002). The sites of neural adaptation induced by resistance training in humans. *Journal of Physiology*.

[24] Jensen JL, Marstrand PCD, Nielsen JB (2005). Motor skill training and strength training are associated with different plastic changes in the central nervous system. *Journal of Applied Physiology*.

[25] Beck S, Taube W, Gruber M, Amtage F, Gollhofer A, Schubert M (2007). Task-specific changes in motor evoked potentials of lower limb muscles after different training interventions. *Brain Research*.

[26] Schieppati M, Trompetto C, Abbruzzese G (1996). Selective facilitation of responses to cortical stimulation of proximal and distal arm muscles by precision tasks in man. *Journal of Physiology*.

[27] Hopkins JT, Ingersoll CD (2000). Arthrogenic muscle inhibition: a limiting factor in joint rehabilitation. *Journal of Sport Rehabilitation*.

[28] Palmieri RM, Tom JA, Edwards JE (2004). Arthrogenic muscle response induced by an experimental knee joint effusion is mediated by pre- and post-synaptic spinal mechanisms. *Journal of Electromyography and Kinesiology*.

[29] Elmqvist LG, Lorentzon R, Johansson C, Fugl-Meyer AR (1988). Does a torn anterior cruciate ligament lead to change in the central nervous drive of the knee extensors?. *European Journal of Applied Physiology and Occupational Physiology*.

[30] Gauffin H, Pettersson G, Tegner Y, Tropp H (1990). Function testing in patients with old rupture of the anterior cruciate ligament. *International Journal of Sports Medicine*.

[31] Urbach D, Nebelung W, Weiler HT, Awiszus F (1999). Bilateral deficit of voluntary quadriceps muscle activation after unilateral ACL tear. *Medicine and Science in Sports and Exercise*.

[32] Wojtys EM, Huston LJ (1994). Neuromuscular performance in normal and anterior cruciate ligament- deficient lower extremities. *American Journal of Sports Medicine*.

[33] Liepert J, Tegenthoff M, Malin JP (1995). Changes of cortical motor area size during immobilization. *Electroencephalography and Clinical Neurophysiology*.

[34] Zanette G, Manganotti P, Fiaschi A, Tamburin S (2004). Modulation of motor cortex excitability after upper limb immobilization. *Clinical Neurophysiology*.

